# Label Free QCM Immunobiosensor for AFB1 Detection Using Monoclonal IgA Antibody as Recognition Element

**DOI:** 10.3390/s16081274

**Published:** 2016-08-11

**Authors:** Özlem Ertekin, Selma Öztürk, Zafer Ziya Öztürk

**Affiliations:** 1 TÜBİTAK, The Scientific and Technological Research Council of Turkey, Marmara Research Center, Genetic Engineering and Biotechnology Institute, Gebze, 41470 Kocaeli, Turkey; selma.ozturk@tubitak.gov.tr; 2 Department of Molecular Biology and Genetics, Gebze Technical University, 41400 Kocaeli, Turkey; 3 Department of Physics, Gebze Technical University, 41400 Kocaeli, Turkey; zozturk@gtu.edu.tr

**Keywords:** QCM immunosensor, IgA monoclonal antibody, aflatoxin immobilization, chemical blocking

## Abstract

This study introduces the use of an IgA isotype aflatoxin (AF) specific monoclonal antibody for the development of a highly sensitive Quartz Crystal Microbalance (QCM) immunobiosensor for the detection of AF in inhibitory immunoassay format. The higher molecular weight of IgA antibodies proved an advantage over commonly used IgG antibodies in label free immunobiosensor measurements. IgA and IgG antibodies with similar affinity for AF were used in the comparative studies. Sensor surface was prepared by covalent immobilization of AFB1, using self assembled monolayer (SAM) formed on gold coated Quartz Crystal, with 1-Ethyl-3-(3-dimethylaminopropyl) carbodiimide/N-hydroxy succinimide (EDC/NHS) method using a diamine linker. Nonspecific binding to the surface was decreased by minimizing the duration of EDC/NHS activation. Sensor surface was chemically blocked after AF immobilization without any need for protein blocking. This protein free sensor chip endured harsh solutions with strong ionic detergent at high pH, which is required for the regeneration of the high affinity antibody-antigen interaction. According to the obtained results, the detection range with IgA antibodies was higher than IgG antibodies in QCM immunosensor developed for AFB1.

## 1. Introduction

Aflatoxins (AF) are secondary metabolites of fungi from *Aspergillus* spp. and can cause acute and chronic toxicity in both humans and animals when ingested [1,2]. They are amongst the most abundant food and feed contaminants, and directives are employed in order to prevent the associated health risks [3]. Internationally accepted precise AF quantification is conducted with laboratory based analytical methods such as HPLC, LC-MS/MS or ELISA, which require expensive, sophisticated equipment and trained staff [1,2]. The availability of rapid and on-site systems for the analysis of AF will both provide better control of AF contamination in food and feedstuff, and also decrease the related analytical costs. Biosensors, which have gained popularity during the past decade, are the most potent solutions towards this goal. A biosensor is defined as a bioanalytical device incorporating a molecular recognition element associated or integrated with a physicochemical transducer [4]. 

Various studies have been conducted in order to develop AF biosensors. Among these studies, immunosensors are widely preferred since they utilize the specificity, selectivity and affinity of the antibodies as sensing elements [5,6,7]. These properties of the antibodies are particularly noteworthy in order to detect analytes that have high toxicity at low concentrations like AF in complex media such as food matrices [8,9]. As a recognition element, the antibody is in close contact with a transducing element that converts the antigen–antibody binding into quantitative electrical or optical signals in biosensors. We used AF as a sensing layer on Quartz Crystal Microbalance (QCM) transducers, and antibodies as recognition elements in an inhibitory immunoassay format. 

A high quality antibody is critical in order to achieve a sufficiently low limit of detection in immunoassays. Antibodies delivered to the sensor surface are expected to interact with very small concentrations of the analyte, particularly in studies conducted with toxins such as AFB1, which has a 5 ng/mL maximum allowable legislative limit for most of the foodstuff in Europe [10]. An AF specific high affinity antibody is crucial for the development of an efficient biosensor. In addition, the detection of small molecule analytes such as AF usually requires various labels like enzymes, nanoparticles, or fluorescent molecules in order to increase sensitivity. 

QCM is a commonly used piezoelectric transducer for immunosensing. It is an extremely sensitive weighing device, which is based on measurement of the change in mechanic resonance of the quartz crystal with changing mass. Quartz crystal (QC) has piezoelectric properties, which, under mechanical stress, produce electrical voltage. On the contrary, when an electric voltage is applied to the crystal, it produces a resonance at a certain frequency, which is affected by the mass on the crystal. According to Sauerbrey’s equation [11] (Δf = −2f_0_^2^Δm/A(ρ_q_μ_q_)^1/2^, where Δf is the counted frequency change (Hz); f_0_ is the fundamental resonance frequency of the quartz oscillator; Δm is the mass change; A is the area of the electrode; ρ_q_ is quartz density; and μ_q_ is the shear stress of quartz), the change in resonant frequency of a QCM is principally based on the mass of adsorbed material on the QCM surface. The shift in resonance frequency (Δf) is proportional to the surface mass (M) of the deposit [8,12]. This mechanism allows label-free detection of analytes. Other label free transducers such as surface plasmon resonance (SPR) sensors rely on optical detection of the molecules on the gold coated sensor chip. Although these systems were proven to be efficient for biosensing, they require relatively more expensive equipment, and the fabrication of sensor strips are more complex and expensive compared to QCM transducers [13].

While working with QCM sensors, structural character of the antibody, which is determined by its isotype, is also an overseen important criterion for signal enrichment. Immunoglobulin G (IgG) antibodies are high affinity antibodies, which are the most abundant isotype in mammals, and accordingly, they are the most easily developed monoclonal antibodies. IgA antibodies are also high affinity antibodies; however, their relatively low abundance make it harder, although possible, to develop as monoclonals. Thus, IgG and IgA antibodies are two notable high affinity choices to be utilized in detection systems. These antibodies differ in their molecular size and the number of antigen binding sites. IgG antibodies have two antigen binding sites with ~150 kDa MW, and IgA has four antigen binding sites with ~340 kDa MW [14]. Higher molecular weight of IgA antibodies can pose a significant advantage while working with QCM sensors, which are actually highly sensitive microbalances. With their higher MW compared to IgG, IgA antibodies are expected to generate a higher frequency shift in QCM transducer systems, which rely on the mass change on the sensor surface, and hence an increase in sensitivity can be expected. QCM based AF sensors utilized signal enhancers such as nanoparticles [10] or magnetic beads [11] and the use of IgA antibodies may compensate this requirement.

Despite IgA antibodies being good candidates, there are few reports regarding their production and use in detection systems [15,16], and there are no records of their use in mycotoxin biosensor development yet. Previously known anti-AF antibodies are mostly IgG isotypes [17,18,19], where a limited number of reports presented monoclonal IgA antibodies [20,21]. However, AF biosensor development literature is based on either polyclonal antibodies [22,23] or monoclonal IgG antibodies [24,25]. 

Another aspect of antibody choice comprises the affinity of the antibody. Although high affinity antibodies have a major role to improve the limit of detection, they require harsh regeneration conditions; such as pH extremes, application of detergents or both. Thus, a sensor surface should be tolerant to different regeneration solutions [26]. Therefore, a properly functionalized surface allowing proper antibody-antigen interaction as well as effective regeneration is crucial. In previous studies, AF was immobilized to the sensor surface via its protein conjugates either by direct adsorption [25,27,28] or using self assembled monolayers (SAM) [29,30] for the development of AF biosensors employing inhibitory immunoassay. However, protein bearing surfaces are labile to harsh regeneration conditions, which may lead to protein denaturation or delocalization preventing successive regeneration cycles. Chemical immobilization of AFB1 to the sensor surface is a means of overcoming challenges related with reproducibility, stability and regeneration. Direct immobilization of AFB1 to a Biacore CM5 sensor chip (Buckinghamshire, UK) was previously described where carboxylated AFB1 derivative AFB1—o carboxymethyloxime (AFB1-oxime) was coupled to amine groups on the CM5 chip [5,31]. 

Surface preparation not only involves antigen immobilization, but also proper blocking of the surface in order to prevent non-specific adsorption of molecules. As a label free transducer, QCM detects not only specific binding, but also nonspecific binding. Therefore, selective binding and efficient blocking of the prepared sensor surface are crucial to avoid interferences with the original signal. This factor is particularly important for analytes with ng/mL level maximum allowable limits, such as AF. Sensor surfaces prepared by the immobilization of analytes via SAM are usually blocked with a nonspecific protein in order to minimize off-target readings [30,32,33]; however, protein blocking is sensitive to harsh regeneration conditions, and chemical blocking is more stable. In the above-mentioned Surface Plasmon Resonance (SPR) sensors, blocking of reactive carboxyl groups was achieved by ethanolamine [5]; however, blocking of unbound reactive amine groups on the AF immobilized surface was not evaluated. Optimization of the coupling reaction, as well as proper blocking of the sensor surface to prevent nonspecific protein binding is also essential for a good performance. 

This study is hitherto the first demonstration of the use of IgA isotype monoclonal antibodies in biosensors. We used a high affinity, AF specific IgA isotype monoclonal antibody as a recognition element to develop a QCM immunosensor. We prepared a protein free sensor surface compatible with the harsh conditions required for the regeneration of the surface from this high affinity antibody. As a result, AF was detected in one step, without any need for enrichment, using an IgA monoclonal antibody based inhibitory immunoassay.

## 2. Materials and Methods

### 2.1. Materials

We used all chemical reagents from Sigma Aldrich, Taufkirchen, Germany with the exception of 1-Ethyl-3-(3-dimethylaminopropyl) carbodiimide (EDC) (Thermo Scientific, Waltham, MA, USA) and Aflatoxin B1 (AFB1) (Fermentek, Jerusalem, Israel). In addition, 5 MHz AT cut quartz crystals with gold-plated electrodes on both sides were purchased from KVG Quartz Crystal Technology GmbH, Neckarbischofsheim, Germany. For the immunoassay, we used in-house developed purified AF-specific murine monoclonal antibodies MAM-D12E2 of IgA isotype and MAM-8G8 of IgG isotype. 

### 2.2. Preparation of the Gold Surface for AF Binding

Gold coated quartz crystals were argon plasma cleaned with Diener Femto plasma cleaner (Ebhausen, Germany) for 3 min at 40 mV prior to surface modification. The coating of quartz was carried out under a sterile hood to avoid contamination. Crystals were soaked in the ethanol solution of 2 mM 11-mercaptoundecanoic acid (MUA) overnight at room temperature. Then, 400 mM EDC was mixed with equal volume of 100 mM N-hydroxysuccinimide (NHS) to obtain an EDC/NHS solution. MUA coated surface was activated with EDC/NHS for either 10 or 15 min. Carboxylic acid moieties of MUA were transformed to amine by incubation of activated surface with 1 M ethylenediamine (EDA), pH:8.5 for 7 min followed by blocking with 1 M ethanolamine (EOA) pH:8.5 for 2 min. 

### 2.3. Chemical Blocking of Activated Surfaces

Unbound amine groups on the crystal surface were blocked with the carboxylic acid in the acetate buffer. Blocking efficiencies of 60 mM acetate buffer, pH:4 and 1 M acetate buffer, pH:4.8 were evaluated. Additionally, 60 mM acetate at pH:4 or pH:7 was activated with EDC/NHS solution for 10 min with 1:5 acetate:EDC ratio, and used for blocking to test the effect of activation on blocking efficiency. The crystals prepared for comparison of 10 or 15 min EDC/NHS activation of MUA prior to amine conversion were blocked with 1 M acetate buffer, pH:4.8. All experiments were carried out with at least three independent replicas.

QCM200 Quartz Crystal Microbalance of Stanford Research Systems, Sunnyvale, CA, USA was used for the measurement of frequency changes. Binding of nonspecific proteins to the prepared the crystal surface was evaluated by sensor measurements with 0.1 mg/mL Bovine Serum Albumin (BSA) in phosphate buffer saline (PBS) (0.1 M KH_2_PO_4_, 150 mM NaCl at pH:7.2). The analysis was conducted at room temperature with a flow rate of 50 µL/min. Frequency changes resulting from protein binding were recorded with in-house developed software. Experiments were conducted with at least 3 replicas. 

### 2.4. AFB1 Immobilization to the Quartz Crystal

For the experiment, 1 mg AFB1 in 400 µL methanol, 2 mg carboxymethyl hydroxylamine HCl (CMO) in 100 µL dH_2_O and 100 µL pyridine were mixed. The reaction proceeded at 100 °C for 3 h followed by 24 h incubation at room temperature. Solvents were evaporated just to dryness under gentle N_2_ flow [34]. Resulting AFB1-oxime was dissolved in PBS to get a 10 mM concentration.

**Figure 1:**
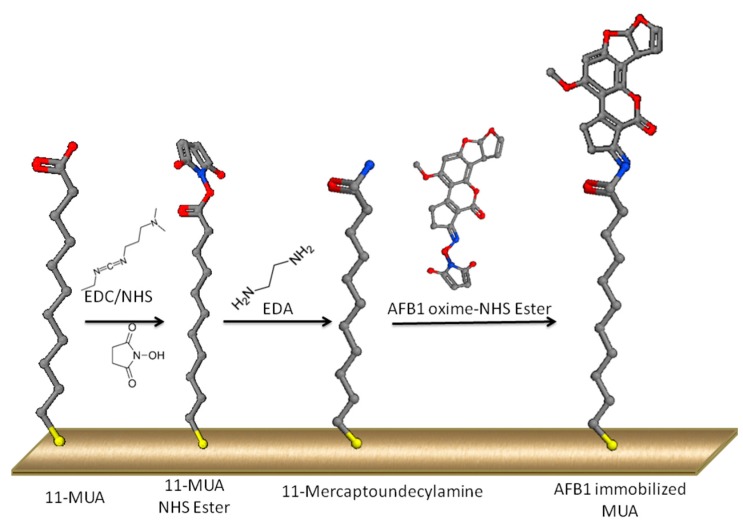
Schematic illustration of AF immobilization strategy to gold coated crystal surface.

In addition, 10 mM AFB1-Oxime was activated by EDC/NHS solution for 10 min with 1:5 AFB1-oxime:EDC ratio. Activated AFB1-oxime was incubated on the amine functionalized surface for 15 min. The surface was blocked with 1 M acetate buffer, pH:4.8 for 10 min. The AF immobilization strategy presented in the current study is summarized in Figure 1.

### 2.5. AFB1 Measurement Procedure

AF specific IgA isotype MAM-D12E2 (IC50_AFB1_: 0.86 ng/mL) and IgG isotype MAM-8G8 (IC50_AFB1_: 0.86 ng/mL) antibodies were used in inhibitory immunoassay with the prepared crystals. 0.1 mg/mL purified MAM-D12E2 or MAM-8G8 antibody was competitively interacted with changing concentrations of AFB1 in PBS for comparative evaluation. MAM-D12E2 antibody was used for the establishment of standard curve for AFB1 in PBS. Antibody-AFB1 mixtures were delivered to the prepared sensor surface at room temperature with 50 µL/min flow rate. Differential frequency shifts resulting from competition of free AFB1 at different concentrations, and surface immobilized AF to bind the antibody were evaluated. The *x*-axis of the sensogams were normalized for alignment using BIAevaluation Software Version 4.1 (Biacore, Buckinghamshire, UK). The experiments were conducted with at least 4 replicas.

Limit of detection (LOD) for the developed sensor was calculated with the formula LOD = (k * sb)/m, where k = 2 for 92.1 confident level, sb is standard deviation of blank, and m is the slope of the linear response curve [35].

### 2.6. Surface Regeneration

Different solutions were used for the regeneration of MAM-D12E2 from sensor surface. HPLC grade water, 0.1 M HCl, 50% Methanol, 50 mM NaOH + 30% ACN, 100 mM Glycine HCl, pH:2, 50 mM NaOH + 0.5% SDS, 50 mM NaOH + 1% SDS and 1.2 mM NaOH + 30 mM EDTA were injected to the sensor surface after antibody binding with 50 µL/min flow rate. Regeneration efficiencies were evaluated by frequency changes in PBS.

### 2.7. Safety Considerations

AFB1 is a class-1 human carcinogen. Therefore, all experiments should be conducted at a biosafety level-2 laboratory with proper protection. Preparation of AF sensors requires extensive handling of the toxin. Liquid waste created during the process was incubated overnight in 1% hypochloride solution and sent to incineration. AF immobilized crystals were cleaned with 1:1:5 solution of hydrogen peroxide (30%), ammonia (25%) and deionized water heated to a temperature of about 75 °C for 3 min [36] and treated with argon plasma cleaner for 3 min at 40 mV prior to use.

## 3. Results and Discussion

### 3.1. Preparation of SAM Functionalized Surface and Blocking Optimization of Free Amine Groups

Reactive groups were introduced to the gold surface by the use of 11-MUA SAM. Carboxylic acid groups of 11-MUA were converted to amine in order to provide binding sites for carboxyl bearing AFB1-Oxime. The reaction involved activation of the carboxyl groups with EDC and NHS prior to the binding of EDA. The surface was activated with EDC/NHS solution for either 10 or 15 min to evaluate the effect of duration on nonspecific binding of proteins to the prepared sensor surface. The frequency changes upon application of 0.1 mg/mL BSA were 4 Hz on 10 min of activated surface, and 9.75 Hz on 15 min activated surface (Table 1). It was shown that EDC/NHS solution, which was used for activation of the carboxyl bearing surface, was incubated on the crystal for 15 min instead of 10 min, nonspecific binding increased two fold. 

EDC/NHS coupling chemistry is one of the most frequently used immobilization methods due to its high coupling yields and robustness. In this reaction, carboxyl groups form an NHS-ester intermediate, which will, in turn, react with the amine groups of the ligand (Figure 1). Despite its high efficiency, this coupling method may result in side-reactions such as esterification of hydroxyl groups as well as cross-activation of ligand carboxyl groups and buffer components with carboxyl groups [37]. Furthermore, NHS-ester intermediate is stable up to several hours at pH:4–5 [38], where our working pH is 4.8 to increase efficiency. One to twenty minutes of activation is recommended for the carboxyl bearing sensor surface, and the duration is a factor affecting the activation level [37]. Limiting the duration of activation both decreases the possibility of side-reactions, and prevents the formation of excess number of NHS-esters, which may lead to nonspecific binding.

Free amine groups on the prepared surface were blocked with carboxylic acid in acetate buffer. Frequency shift resulting from 0.1 mg/mL BSA solution was used to demonstrate the blocking efficiency of the surface. Amine activated surface was treated with 60 mM acetate buffer, pH:4 or 1 M acetate buffer, pH:4.8 to evaluate their blocking efficiencies. Additionally, carboxylic acid groups of 60 mM acetate buffer were activated with EDC/NHS, and used as blocking agent. 

**Figure 2:**
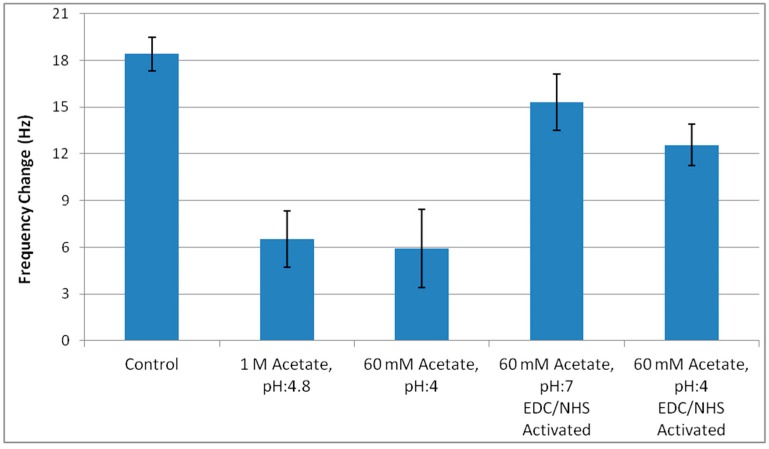
Blocking optimization on amine activated surface. Non-specific protein binding to sensor surface after blocking with 1 M acetate buffer, pH:4.8, 60 mM acetate buffer, pH:4 or EDC/NHS activated 60 mM acetate buffer. Error bars represent standard errors.

Figure 2 presents nonspecific protein binding after different blocking conditions. In control crystal, the surface bears many unblocked highly reactive amine groups. Application of acetate buffer, either 60 mM, pH:4 or 1 M pH:4.8, significantly reduced nonspecific binding to the crystals. Nevertheless, when a carboxyl group of acetate was activated with EDC/NHS solution in order to improve the blocking efficiency, the blocking property of the solution completely vanished. This result was strongly correlated with the previous finding that the duration of EDC/NHS activation of MUA increased the nonspecific reactivity of the surface. We consider the reason for the extreme reactivity is the retreatment of the surface with EDC/NHS solution. 

Application of activated acetate can be considered as a simulation of AF-oxime binding, so we cannot avoid the exposure of the surface to EDC/NHS solution. Considering the better reproducibility and stability of the surface blocked with 1 M Acetate buffer, pH:4.8, this condition was chosen for blocking of AFB1-oxime bound crystals.

### 3.2. Inhibitory Immunoassay Using IgA or IgG Antibodies

**Figure 3:**
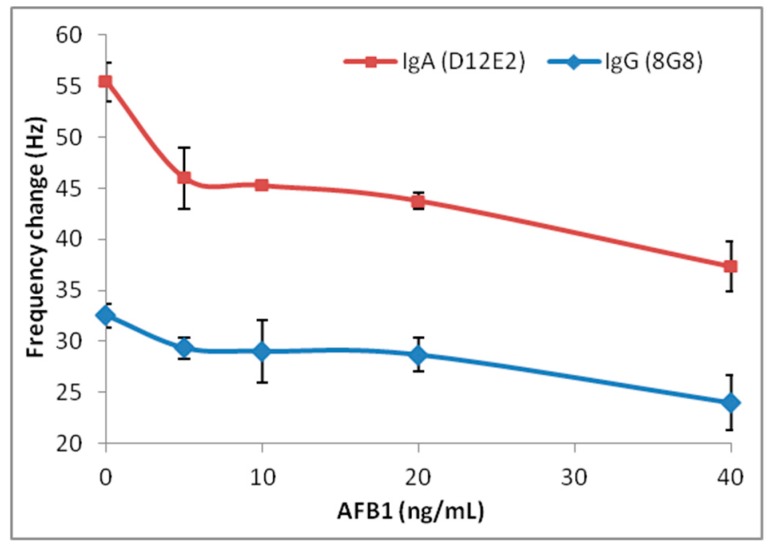
Inhibitory AFB1 analysis with MAM-8G8 and MAM-D12E2 antibodies using AFB1 immobilized sensor chip (0–40 ng/mL AFB1). Error bars represent the standard errors.

Changing concentrations of AFB1 (5 ng/mL–40 ng/mL) was mixed with equal volume of 0.1 mg/mL IgA isotype MAM-D12E2 antibody or IgG isotype MAM-8G8 antibody with similar affinity to AFB1. The mixture was delivered to the AF immobilized sensor surface, and frequency shifts were recorded (Figure 3). Maximal frequency shifts observed in control samples with no AFB1 competition were different in two antibodies as an indication of antibody structure. IgG isotype antibody (150 kDa) produced 32 Hz and IgA isotype MAM-D12E2 antibody (340 kDa) produced 55 Hz control sensor response. When we examined the frequency changes in AFB1 competition samples with respect to control samples, we showed that higher control sensor response was correlated with greater sensor signals (Table 2). MAM-D12E2 showed three times as much frequency difference with respect to control at 5–20 ng/mL AFB1 competition, and twice as much frequency difference at 40 ng/mL AFB1 competition when compared to MAM-8G8. The sensor was developed using the IgA antibody due to its broader AFB1 competition range. 

### 3.3. Optimization of Sensor Surface Regeneration

**Figure 4:**
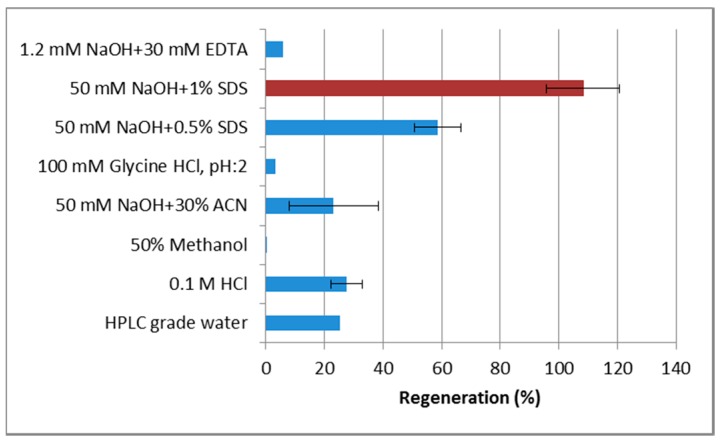
Regeneration of AFB1 immobilized sensor surface after MAM-D12E2 application using different solutions. Results are presented as the percentage of the antibody recovered from the surface, calculated with binding and regeneration frequency shifts.

Recovery of MAM-D12E2 antibody from sensor surface using different regeneration solutions is presented in Figure 4. MAM-D12E2 antibody binding can be fully recovered with 50 mM NaOH and 1% SDS. The antibody could not be regenerated using solutions that disrupt ionic bonds, but efficiently regenerated with a strong ionic detergent. This result is the indication of a hydrophobic interaction of the antibody with AF.

**Figure 5:**
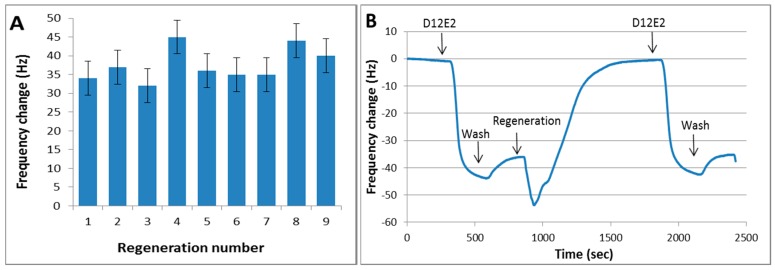
Regeneration of AF immobilized sensor surface. (**A**) Sequential regeneration of the sensor surface with 50 mM NaOH and 1% SDS. Graph shows sensor response as frequency change upon delivery of 0.1 mg/mL MAM-D12E2 antibody after each regeneration; (**B**) Sensogram showing a typical regeneration cycle with MAM-D12E2 antibody.

Reproducibility of regeneration was tested with sequential injections of 0.1 mg/mL MAM-D12E2 antibody to the regenerated surface. The surface could be regenerated at least nine times without significant loss of performance (Figure 5). Similar surfaces were shown to be regenerated up to 75 times with antibodies that can be regenerated at milder conditions such as 10 mM NaOH application [31]. However, MAM-D12E2 is a very high affinity antibody that can only be regenerated using strong ionic detergents in addition to high pH, and at least nine sequential regenerations could be achieved through the use of the protein-free sensor surface. We observed that the number of regeneration cycles could be improved when we wash the surface with water after the application of regeneration solution, before PBS injection. 

### 3.4. QCM Based Detection of AFB1

**Figure 6:**
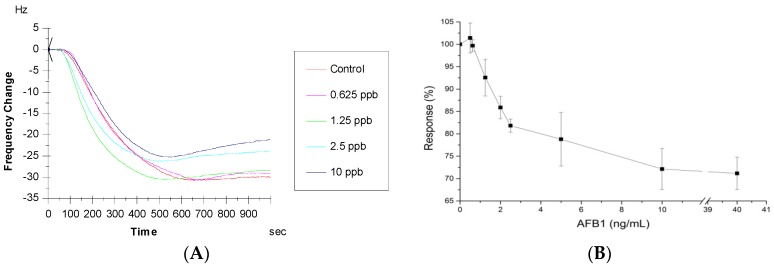
AFB1 sensor response with IgA antibody (**A**) Sensograms showing the sensor response at different concentrations of AFB1; (**B**) Sensor response curve for AFB1 detection. Error bars represent standard errors.

After the determination of the antibody to be used as a sensing element and optimization of the assay conditions including regeneration and competition, we generated a standard curve for AFB1 detection using MAM-D12E2 antibody. In the work conducted for the choice of sensing element, high concentrations of AFB1 were chosen in order to be able to evaluate the working competition range for each antibody. The sensograms showing the frequency shifts upon application of different AFB1 concentrations in inhibitory immunoassays is presented in Figure 6A. Dose response curve showing the sensor response to different AFB1 concentrations is shown in Figure 6B. The results show that the surface is responsive to the inhibition assay conducted with IgA isotype MAM-D12E2 antibody and AFB1. The limit of detection was shown to be 1.625 ng/mL with 1.25 ng/mL–10 ng/mL detection range. The linear range of the curve was 0.625–2.5 ng/mL (R^2^ = 0.998). Sensitivity calculated from the slope of the linear curve indicated a 9.6% change for 1 ng/mL AF. We presume two possible causes about the source of variation in the data. The initial reason for this variation may be the lack of temperature control of the experimental setup, where temperature fluctuations affect A/T cut crystals 8 Hz/1 °C in aqueous phase [39]. The second reason we propose is the fact that the data could not be normalized according to the nonspecific binding since the experiments were conducted in a single oscillator device that did not allow parallel experiments. 

Different maximum allowable AF levels for different feed products established by different countries and organizations. The lowest maximum allowable AFB1 levels set by EU go down to 2 µg/kg for products intended for the consumption of infants. The highest limits are applied for foodstuff such as pistachio and almonds, which is 12 µg/kg [3]. Therefore, the developed sensor has the potential to be used for the detection of AF in foodstuff.

To the authors’ knowledge, there are two previous studies using AF immobilized surface in QCM immunosensor development for AF detection, both of which utilized murine monoclonal IgG antibodies. In these studies, AFB1-BSA conjugate was immobilized to the gold surface, and detection required the use of secondary antibodies. The first study used an enzyme labeled secondary antibody for electrochemical QCM measurement, and had a detection range of 0.01–10 ng/mL [30]. The second study used gold nanoparticle labeled secondary antibodies, and achieved a detection range of 0.1–100 ng/mL [40]. Other work employing a competitive immunoassay strategy used either monoclonal IgG antibodies or polyclonal antibodies, and utilized optical or electrochemical transducers. Among the label-free optical sensors developed with the same strategy, there are two SPR studies using a similar surface preparation approach. One study used monoclonal IgG antibodies, and could achieve a detection range of 0.2–10 ng/g with prior concentration of AFs using IACs [5], and the other used multimeric recombinant antibody fragments, and had 0.2–24 ng/mL detection range [31]. Other optical AF sensors utilized either fluorescent [25,41] or chemiluminescent [27] labels for limits of detection ranging from 0.1 to 1.5 ng/mL. Therefore, the present study was the first development of competitive AF immunosensor development without using any kind of labels or tags, which was possible with the use of an IgA isotype antibody.

## 4. Conclusions

AF was immobilized on to gold coated QCM crystal functionalized via SAM as sensing layer for the direct determination of AF in samples. Our results indicated that nonspecific reactivity of the surface increases with increasing duration of EDC/NHS application to the surface. EDC/NHS activation is commonly used in sensor development studies, and the duration of activation should carefully be optimized to obtain minimal exposure of the surface. On the other hand, nonspecific binding to the surface can be minimized by careful choice of blocking reagents. We used a carboxyl group bearing acetate buffer to block unreacted amine groups on the surface. Our results showed that 1 M acetate buffer, pH:4.8 provided a good blocking efficiency. The prepared surface was regenerated for at least nine sequential analyses with strong detergent containing regeneration solution, which would disrupt conventionally used protein containing surfaces.

**Figure 7:**
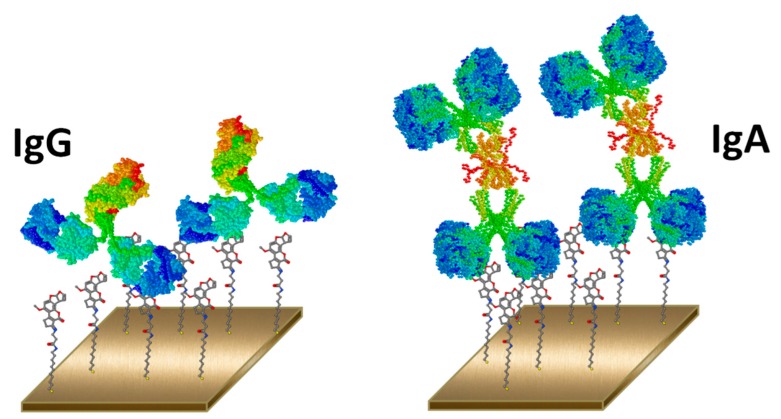
Use of different antibody isotypes in sensor development.

We developed a QCM immunosensor for AFB1 detection using monoclonal IgA antibody, and showed its advantage over a conventional AF specific IgG antibody with similar affinity to AFB1. In this hitherto first study of sensor development with IgA antibodies, we compared frequency shifts observed upon AFB1 competition for both antibodies. A three times wider working frequency range was observed with MAM-D12E2, a monoclonal IgA antibody which has twice molecular weight when compared to IgG isotype MAM-8G8, in the inhibitory immunoassay. In the immunoassay development, we did not use any labels like magnetic beads or dendrimeric material for signal enrichment. AF was detected with monoclonal IgA antibody based inhibitory immunoassay in only one step. We showed that higher molecular weight of IgA antibodies contributed to a higher sensitivity, and provided a natural signal enrichment for QCM immunosensor development (Figure 7).

The results showed that the IgA antibodies provide sufficient affinity and signal enrichment in QCM immunosensor development, and this concept can be implemented for different target analytes for the development of mass sensitive sensors.

## Figures and Tables

**Table 1:** Effect of different durations of EDC/NHS activation on non-specific protein binding to the MUA coated QCM crystals.

EDC/NHS Application Time (min)	Frequency Change upon 0.1 mg/mL BSA Application (Hz)
10	4.0 ± 1.4
15	9.75 ± 1.8

**Table 2:** The frequency difference between control and competition samples for MAM-8G8 and MAM-D12E2.

AFB1 (ng/mL)	MAM-8G8 (0.1 mg/mL)	MAM-D12E2 (0.1 mg/mL)	Fold Difference (MAM-D12E2/MAM-8G8)
5	3.2 Hz	9.4 Hz	3.0
10	3.5 Hz	10.2 Hz	2.9
20	3.8 Hz	11.7 Hz	3.0
40	8.5 Hz	18.1 Hz	2.1
